# Reservoir characteristics and logging evaluation of gas−bearing mudstone in the south of North China Plain

**DOI:** 10.1038/s41598-020-65325-1

**Published:** 2020-05-29

**Authors:** Liang Liu, Heping Pan, Zhenzhou Lin, Shihui Zhang, Zhen Qin, Jianwei Li, Guoshu Huang, Lei Wang, Dong Li

**Affiliations:** 1 Institute of Geophysics and Geomatics, China University of Geosciences (Wuhan), Wuhan, 430074 China; 2 Institute of Geophysical and Geochemical Exploration, CAGS, Langfang, 065000 China; 3 School of Geophysics and Measurement-Control Technology, East China University of Technology, Nanchang, 330013 China; 4 Well Logging Company of Sinopec North China Petroleum Engineering Company, Zhengzhou, 450007 China

**Keywords:** Environmental sciences, Geology, Engineering

## Abstract

Mudstone is very similar to shale except it lacks sheet bedding. Shale gas is widely concerned and successfully exploited commercially in the world, while gas-bearing mudstone is rarely paid attention. To evaluate the reservoir characteristics and exploitation potential of gas-bearing mudstone, a total of 127 mudstone samples from the Shanxi formation were tested by X-ray diffraction (XRD), scanning electron microscope (SEM), gas content, etc., and the qualitative identification and quantitative evaluation of gas-bearing mudstone reservoirs were performed on four wells using the logging curve overlay method and reservoir parameter calculation equations. The results showed that: (1) the average total gas content of core measurement is 1.81 m^3^/t, and the total content of brittle minerals is 44.2%, which confirms that mudstones can also have good gas content and fracturing performance; (2) logging evaluation the average thickness of gas-bearing mudstone is 55.7 m, the average total gas content is 1.6 m^3^/t, and the average brittleness index is 38.1%, which indicates that the mudstone of Shanxi formation in the study area is generally gas-bearing and widely distributed. All the results reveal that gas-bearing mudstone with block bedding has the same exploitation potential as shale with sheet bedding,which deserves more attention.

## Introduction

Mudstone and shale are very similar. The difference is that the mudstone lacking the laminations of shale, which easily forms a block shape when it is broken, while shale has bedding structure, which splits easily into thin flat layers. Although both are commonly referred to as shale in a broad sense, the source rock is mainly composed of mudstone and siltstone^1,2^. In order to comply with the actual characteristics of the formation in the study area and avoid confusion in concept, the concept of mudstone in this paper refers to relatively homogeneous mudstone with block bedding, including silt mudstone, and the shale refers to strong heterogeneous mudstone with sheet bedding, including siltstone.

There has been a lot of research on shale gas worldwide. The famous shale gas production areas include the Barnett, Fayetteville, Haynesville and Marcellus in the United States, the Montney and Horn River in Canada, and the Fuling and Changning in China, etc^3–10^. It is a fossil energy stored in unconventional reservoirs and has great exploration prospects^11–14^. For shale gas reservoirs, the shale itself is both a source rock and a reservoir^15^. Shale gas is mainly stored in two forms, one is in the free state in the cracks and intergranular pores, the other is adsorbed on the surface of kerogen and clay particles. In addition, there may be very little shale gas dissolved in the bound water, kerogen and bitumen^15–19^. Gas-bearing mudstones and shale have similar reservoir characteristics, and many shale gas production areas in China are often interbedded with mudstone and shale rather than single lithology of shale. Therefore, many chinese scholars have studied them as integration and defined them as mud-shale or mudstone-shale reservoirs^20–24^.

There are mud-shale reservoirs in the Shanxi and Taiyuan formations in the south of the North China Plain. These reservoirs have a stable gas production of more than 3000 m^3^/d in some wells, which indicating good prospects for mud shale gas exploration and development^25–27^. However, according to the data of cores and drilling cuttings, the mudstones of Shanxi formation of four wells A1, A2, A3 and A4 in the study area of this paper have no bedding. For this mudstone formation, it has been evaluated as a cap rock and a source rock for a long time, and it is rarely considered as a reservoir^27^. In order to find out whether mudstone formation has the same mining potential as shale or mud-shale formation, this paper focuses on the mudstone of the Shanxi formation of the four wells A1, A2, A3, and A4 in the southern North China Plain. Firstly, a total of 127 core samples of Shanxi formation mudstone in well A2 were tested by X-ray diffraction (XRD), electron microscopy (SEM), total organic carbon content (TOC), vitrinite reflection (R_O_) and gas content respectively, and their mineral characteristics, reservoir space types, physical properties, hydrocarbon generation capacity and gas content were summarized. Then, based on the well logging data, the well logging response characteristics of mudstone formations with different gas contents were analyzed. Finally, qualitative identification of gas-bearing mudstone reservoirs and quantitative evaluation of mudstone formation sedimentary environment, gas content, porosity, permeability, and brittleness index were performed using well logging methods.

## Geological Setting

After the Indosinian movement, the mainland of China was dominated by intracontinental deformation. Due to the collision and compression of the Qinling-Dabie orogenic belt, since the Late Triassic, the depression began to appear in the north of South China, resulting in the fault between wells A3 and A4^28,29^. During the development of the depression, the Permian in the south of North China Plain developed four sets of source rocks from the bottom to the top, including the Taiyuan formation, the Shanxi formation, the Xiashihezi formation and the Shangshihe formation. Coal measures mudstone and carbonaceous mudstone are the main source rocks, followed by coal. The argillaceous source rocks have reached the maturity stage as a whole. The relatively high abundance of organic matter is mainly in the Taiyuan formation, Shanxi formation and Xiashihezi formation. The source rocks are mainly type III (humic type), which has good hydrocarbon generation potential^27^.

**Figure 1:**
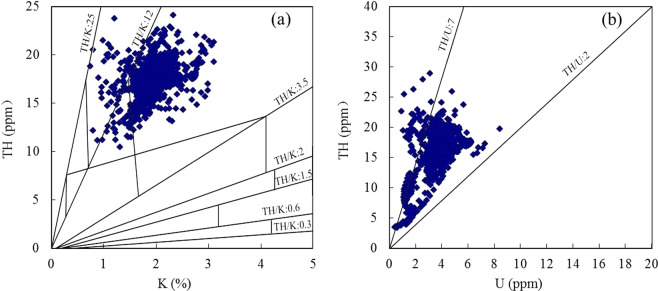
Location of 4 wells in the study area.

Figure 1 shows the geographical location of the study area, including the location of 4 wells. The dark mudstone and shale of the Permian Shanxi formation are widely distributed in the study area, with a total thickness of 30–100 meters^26^. The lithology of Shanxi formation mainly includes mudstone, silty mudstone, shale, siltstone, fine sandstone and coal seam. Mudstone and shale of Shanxi and Taiyuan formations are the main gas producing reservoirs in the study area^30^.

## Methods

### Qualitative evaluation

**Figure 2:**
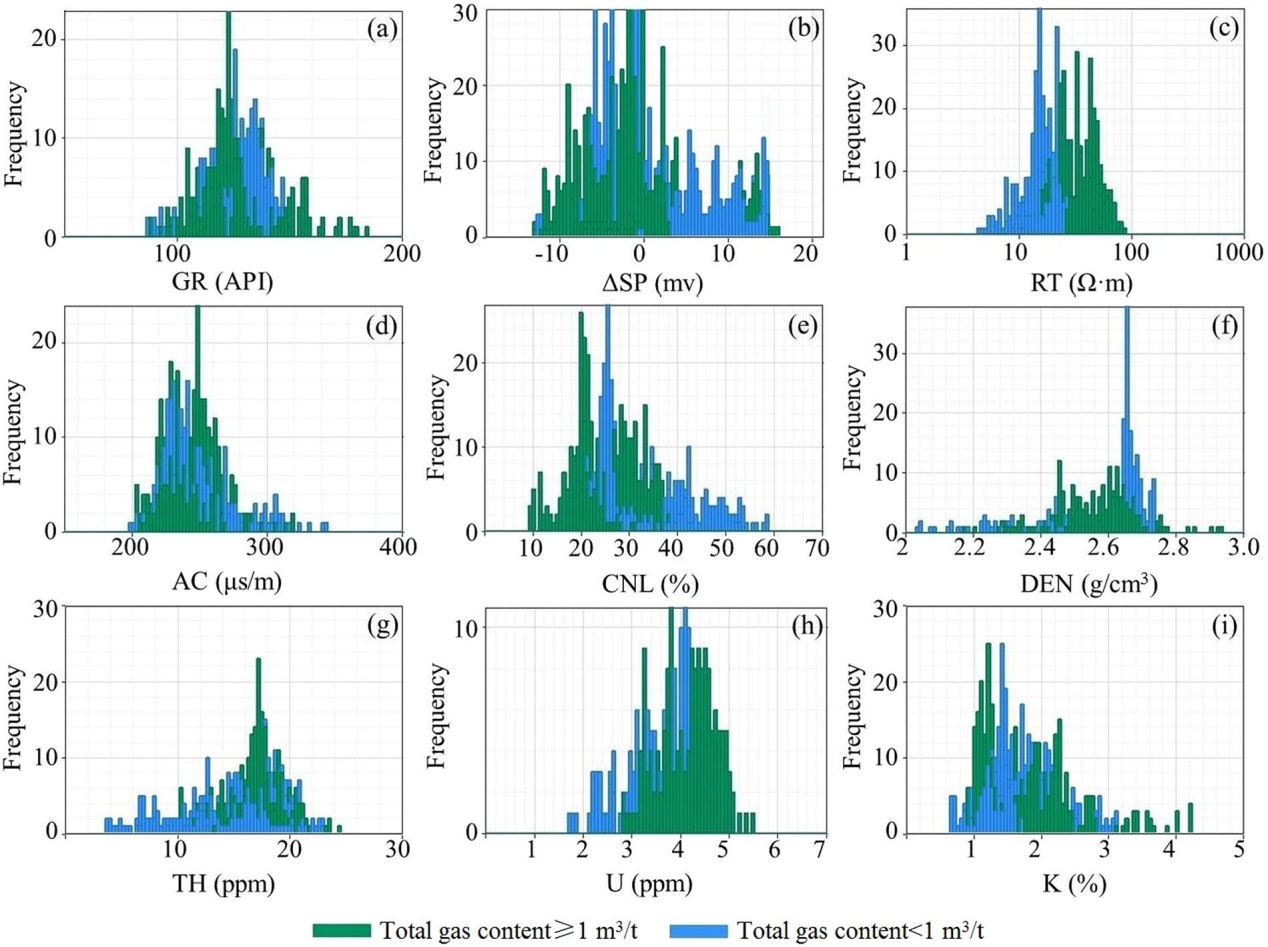
Qualitative evaluation of the relative gas content of mudstone in Shanxi formation of well A4.

It is a fast and effective method to qualitatively evaluate the relative gas content of mudstone reservoir in different depth by using resistivity and neutron or uranium and neutron overlap. The method is to find a depth that makes its overlapping area relatively minimum. At this depth, adjust the left and right boundary values of resistivity and neutron or uranium and neutron to make them coincide, which means that the gas content at this depth is minimum, so the larger the overlapping area at other depths means the better the gas content. The third track in Fig. 2 shows the overlap of resistivity and neutrons in well A4, where the resistivity and neutron optimization boundary values are 2–200 Ω•m and 0–130%, respectively. This is based on the standard of selecting the place with the smallest overlapping area of XX80 m, which means that the gas content here is the smallest. The overlap area at other depths will vary with the gas content of the mudstone formation. The evidence that the minimum gas content of XX80 m is the total hydrocarbon content of the fifth track, which is obtained from gas logging during drilling and can be used as a reference for the gas content. It is worth noting that although the total hydrocarbon content only represents the free gas content in the wellbore, the core measurement data shows that the free gas content is in direct proportion to the total gas content. To some extent, the depth with high free gas content also means high total gas content.

Figure 2 shows that the total gas content of mudstone below the coal seam is higher than that above. The total hydrocarbon content curve also proves this. Similarly, the fourth track in Fig. 2 is the overlap of uranium and neutron. The optimum boundary value of resistivity is 7−0 ppm, and the neutron is 80−0%. It has the same qualitative evaluation results of gas content as the resistivity and uranium overlap. It is worth noting that this method is only suitable for the comparison of gas content at different depths in the same well. It is a method for quickly identifying the relative size of gas content. In other wells, the left and right boundary values need to be transformed to achieve the best evaluation. In addition, it must be noted that this is the left or right boundary value set for mudstone formation, so the overlapping area of sandstone must be ignored, which is meaningless regardless of its size.

### Quantitative evaluation

**Figure 3:**
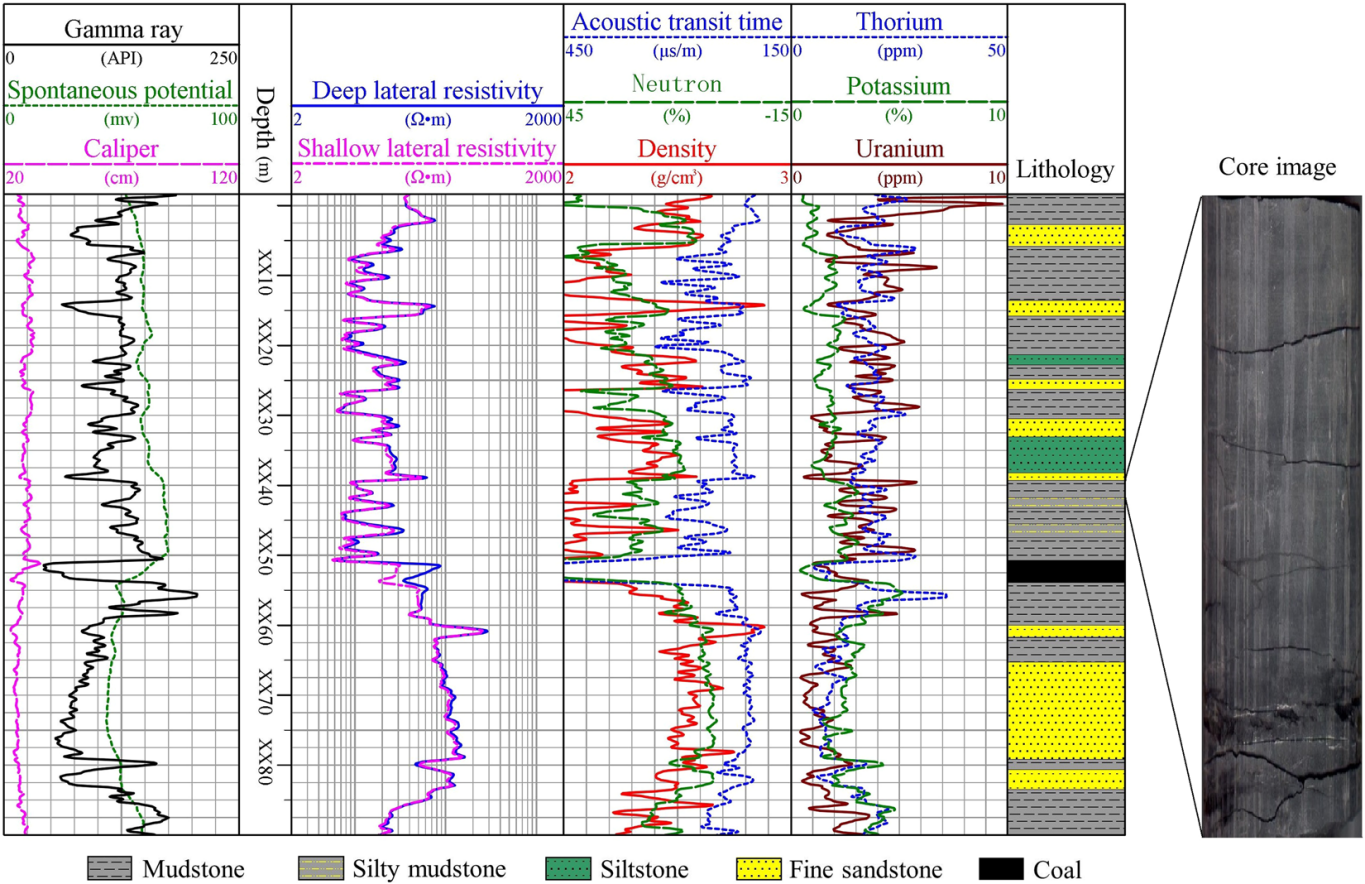
Logging evaluation of total gas content of mudstone in Shanxi formation of four wells.

The reservoir characteristics of gas-bearing mudstone are analyzed by the collected core measurement data, and the logging response characteristics are analyzed by the response values of various logging methods. Logging evaluation is mainly for estimating the porosity, permeability, gas content and brittleness index of mudstone formation. These calculation formulas are shown in Table 1, which is based on the calculation methods of other scholars and obtained by regression based on experimental data in the study area^22,31^. In order to more clearly show the change in total gas content between the four wells, Fig. 3 shows the color filling of the difference in total gas content.

**Table 1:** Equation for quantitative evaluation of mudstone reservoir.

Equation	Unit	Parameter description
Porosity = 0.0453 *AC*−8.0141	%	AC = acoustic transit time, μs/m.
Permeability = 0.0032 *EXP*(0.8074 *ϕ*)	mD	ϕ = Porosity, %.
Adsorbed gas content = 0.1132 *TOC* + 0.0136 *V*_*sh*_ + 0.2379;	m^3^/t	TOC = Total organic carbon content, %; V_sh_ = Clay mineral content, %; GR = natural gamma ray, API; RT = deep lateral resistivity, Ω•m.
TOC = 0.02 *GR*·*LOG(RT)*−1.5628;		
V_sh_ = (2^*GC·SH*^ − 1)/(2^*GC*^ − 1);		
SH = (*GR − GR*_*min*_)/(*GR*_*max*_ *− GR*_*min*_)		
Free gas content = 1.4219 *Adsorbed gas content* + 0.2747	m^3^/t	
Total gas content = *Free gas content* + *Adsorbed gas content*	m^3^/t	
Brittleness index = (*Quartz* + *Feldspar* + *Calcite* + *Dolomite*)/*Total mineral content*;	%	
Feldspar = −0.02761*AC*−0.37232*CNL*−12.1707*DEN* + 37.51644;		
Calcite = −0.04584*AC* + 0.289536*CNL*−4.23927*DEN* + 16.63153;		
Dolomite = 0.002142 *AC*−0.04989*CNL*−0.42518*DEN* + 6.098136;		
Pyrite = 0.013366 *AC*−0.04442*CNL* + 8.707649*DEN*−10.3304;		
Quartz = 100 − *Porosity*−*Clay mineral*−*Feldspar*−*Calcite*−*Dolomite* − *Pyrite*;		
Total mineral content = *Clay mineral* + *Quartz* + *Feldspar* + *Calcite* + *Dolomite* + *Pyrite*		
Constants	GC = 2; GR_min_ = 30 API; GR_max_ = 200 API.

## Results and Discussion

### Characteristics of gas-bearing mudstone reservoirs

#### Thickness of gas-bearing mudstone reservoir

**Figure 4:**
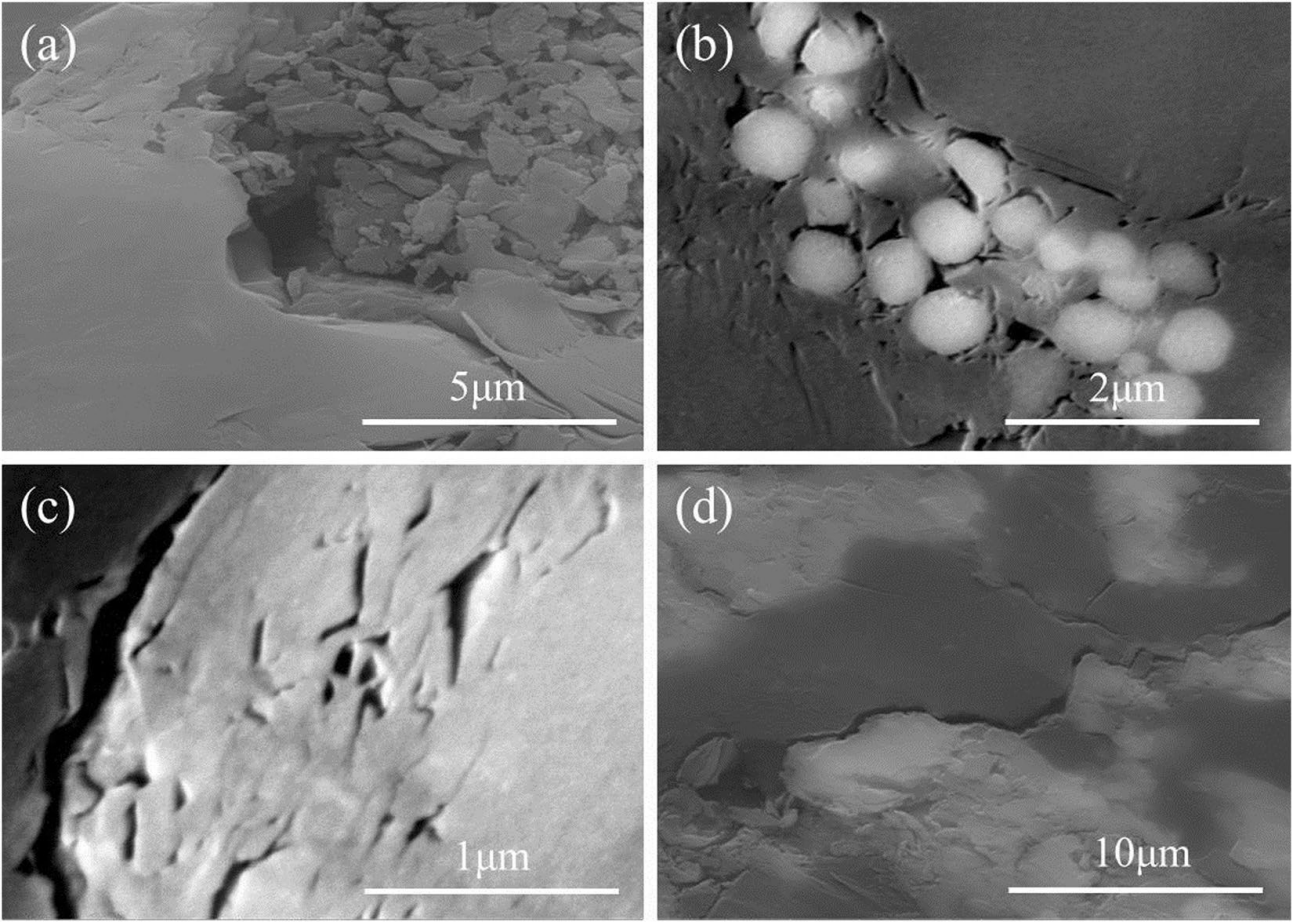
Average values of various characteristics of gas bearing mudstones in Shanxi formation of four wells. Response characteristics from logging data (**a**). Mineral characteristics (**b**), geophysical and geochemical characteristics (**c**) from core measurements. Where GR is the natural gamma ray, RT is the deep lateral resistivity, AC is the acoustic transit time, DEN is the density, CNL is the neutron, K is the potassium, TH is the thorium, U is the uranium, H is the thickness, TOC is the total organic carbon content, and Ro is the vitrinite reflectance.

The gas−bearing mudstones of the Shanxi formation in the south of the North China Plain are distributed above and below the coal seam. Table 2 shows the thickness of the mudstone reservoir after deducting the thickness of siltstone and fine sandstone interlayers. In the four wells, the thickness of the mudstone reservoir above the coal seam ranges from 23.5 to 45 m with an average of 32.3 m. The thickness distribution below the coal seam is 11–29.5 m with an average of 23.5 m. The average thickness of the mudstone reservoir in the Shanxi formation of four wells is 55.7 m (Fig. 4(a)), which means that the mudstone reservoir has the value of mining in terms of thickness.

**Table 2:** Thickness statistics of gas-bearing mudstone reservoirs in Shanxi Formation of four wells.

Borehole ID	Thickness above the coal seam (m)	Thickness below the coal seam (m)	Total thickness (m)
A1	23.5	28.0	51.5
A2	32.0	11.0	43.0
A3	45.0	29.5	74.5
A4	28.5	25.4	53.9
Average	32.3	23.5	55.7

#### Mineral characteristics of gas-bearing mudstone reservoirs

**Figure 5:**
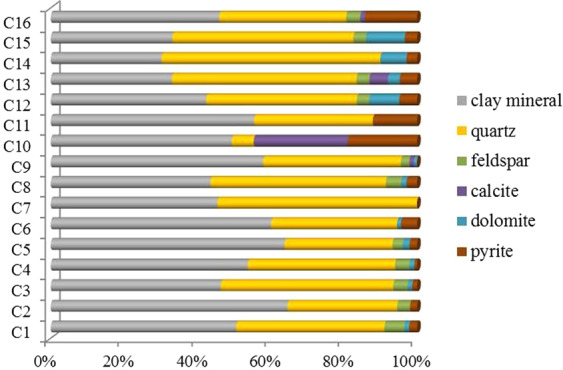
Mineral content statistics of mudstone samples in well A2.

Through the analysis of drilling cuttings, the lithology of the gas-bearing mudstone reservoirs in the Shanxi Formation is mainly dark black mudstone and silty mudstone. X-ray diffraction (XRD) analysis of 16 mudstone samples from well A2 shows that the mineral composition of mudstone is clay mineral (25.3–64%), quartz (5.8–54.5%), feldspar (0–5.3%), calcite (0–24.8%), dolomite (0–10.4%) and pyrite (0–18.3%) (Fig. 5). The average proportion of these minerals is 47.8% of clay minerals, 38.9% of quartzite, 2.9% of feldspar, 2.6% of calcite, 2.7% of dolomite and 5.1% of pyrite, respectively (Fig. 4(b)). Brittle minerals include quartz, calcite and dolomite, the sum of which is 44.2%. This indicates that mudstone reservoirs are fracturable.

#### Reservoir space types and physical properties

**Figure 6:**
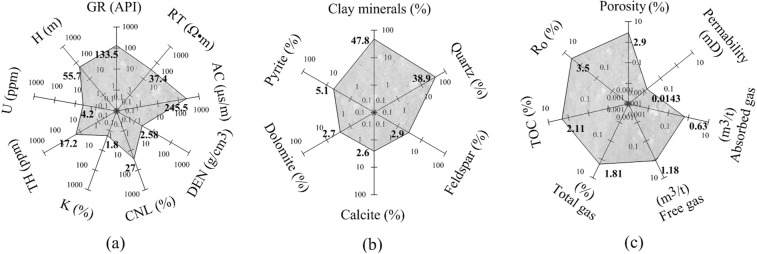
SEM images of intergranular pore (**a**,**b**), intragranular pore (**c**) and microcrack (**d**) in the gas−bearing mudstone reservoir of well A2.

The gas reservoir space in mudstone is mainly composed of mineral matrix pore and micro−fracture. Figure 6 shows the SEM images of the intergranular pore, intragranular pore and micro-fracture in the core samples of the A2 well mudstone reservoir. The measurement results of 14 cores show that porosity ranges from 0.3% to 4.3% with an average of 2.9% and permeability ranges from 0.0045 mD to 0.0336 mD with an average of 0.0143 mD (Fig. 4(c)). Therefore, the gas-bearing mudstone reservoir has extremely low porosity and permeability.

#### Mudstone hydrocarbon generation capacity and gas content

Vitrinite is a type of kerogen. Vitrinite reflectance (R_o_) can be used as an indicator of maturity and thermal evolution of organic matter. When R_o_ > 2.0%, the mudstone reservoir belongs to the organic overmature phase, and the kerogen releases dry gas^31,32^. The data from the 28 mudstone core samples of the Shanxi Formation show that the R_o_ ranges from 3.0% to 3.8% and the average is 3.5% (Fig. 4(c)), which proves that the mudstone reservoir has a high thermal evolution of organic matter. To the extent, the kerogen release is almost entirely gas. The TOC is an organic abundance evaluation index widely used in the world^33,34^. The TOC of 48 samples in the Shanxi Formation ranges from 0.16% to 30.76% and averages 2.11%. The R_o_ and TOC indicate that the Shanxi Formation mudstone has good hydrocarbon generation capacity.

The gas content data measured by 21 cores show that the adsorbed gas content ranges from 0.15 m^3^/t to 1.64 m^3^/t with an average of 0.63 m^3^/t, and the free gas content ranges from 0.42 m^3^/t to 2.45 m^3^/t with an average of 1.18 m^3^/t. The content of free gas is nearly twice that of adsorbed gas. The total gas content ranges from 0.71 m^3^/t to 3.7 m^3^/t with an average of 1.81 m^3^/t (Fig. 4(c)).

#### Well log response characteristics

**Figure 7:**
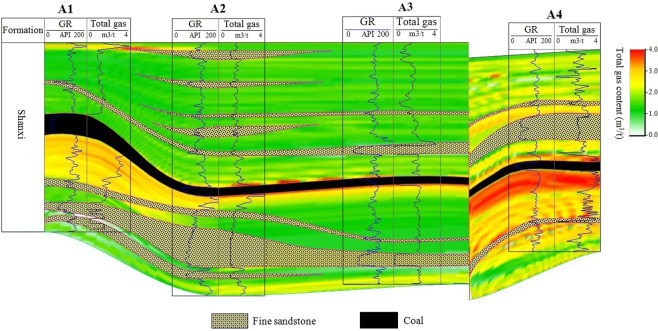
Well logging response of the Shanxi formation in the well A2.

Figure 7 shows an example of the response characteristics of all logging methods in Shanxi formation of well A2. Table 3 summarizes the well log responses of the Shanxi Formation mudstone reservoirs in four wells. Compared with sandstone, the mudstone natural gamma ray, acoustic transit time, neutron, thorium, uranium and potassium increase, the resistivity and density decrease, and the amplitude of spontaneous potential decreases to make it approximately a straight line, which is called shale SP baseline^35^.

**Table 3:** Well log response statistics of mudstone and sandstone reservoirs in Shanxi Formation with 4 wells, where ΔSP is the difference in the amplitude of the spontaneous potential between the shale SP baseline and the mudstone or sandstone reservoir.

Log data	Mudstone		Sandstone	
	Distribution range	Dominant distribution range	Distribution range	Dominant distribution range
GR (API)	85.0–185.2	116.0–140.0	50.2–100.1	80.0–90.0
RT (Ω·m)	4.1–90.2	15.0–50.0	20.0–180.7	40.0–80.0
AC (μs/m)	198.0–342.4	220.0–258.0	210.0–290.0	230.0–270.0
CNL (%)	9.0–59.2	20.0–34.0	8.0–24.0	12.0–16.0
DEN (g/cm^3^)	2.03–2.93	2.44–2.69	2.41–2.72	2.52–2.70
K (%)	0.6–4.2	1.0–2.3	0.7–1.5	0.9–1.3
TH (ppm)	3.6–24.5	12.5–21.2	8.0–14.0	10.0–12.0
U (ppm)	1.6–5.6	3.0–4.8	1.5–3.0	2.0–2.5
ΔSP (mv)	−12.7–16	−6.0–2.0	−25.0– −2.0	−12.0– −7.0

Less than the shale SP baseline is negative, greater than shale SP baseline is positive.

**Figure 8:**
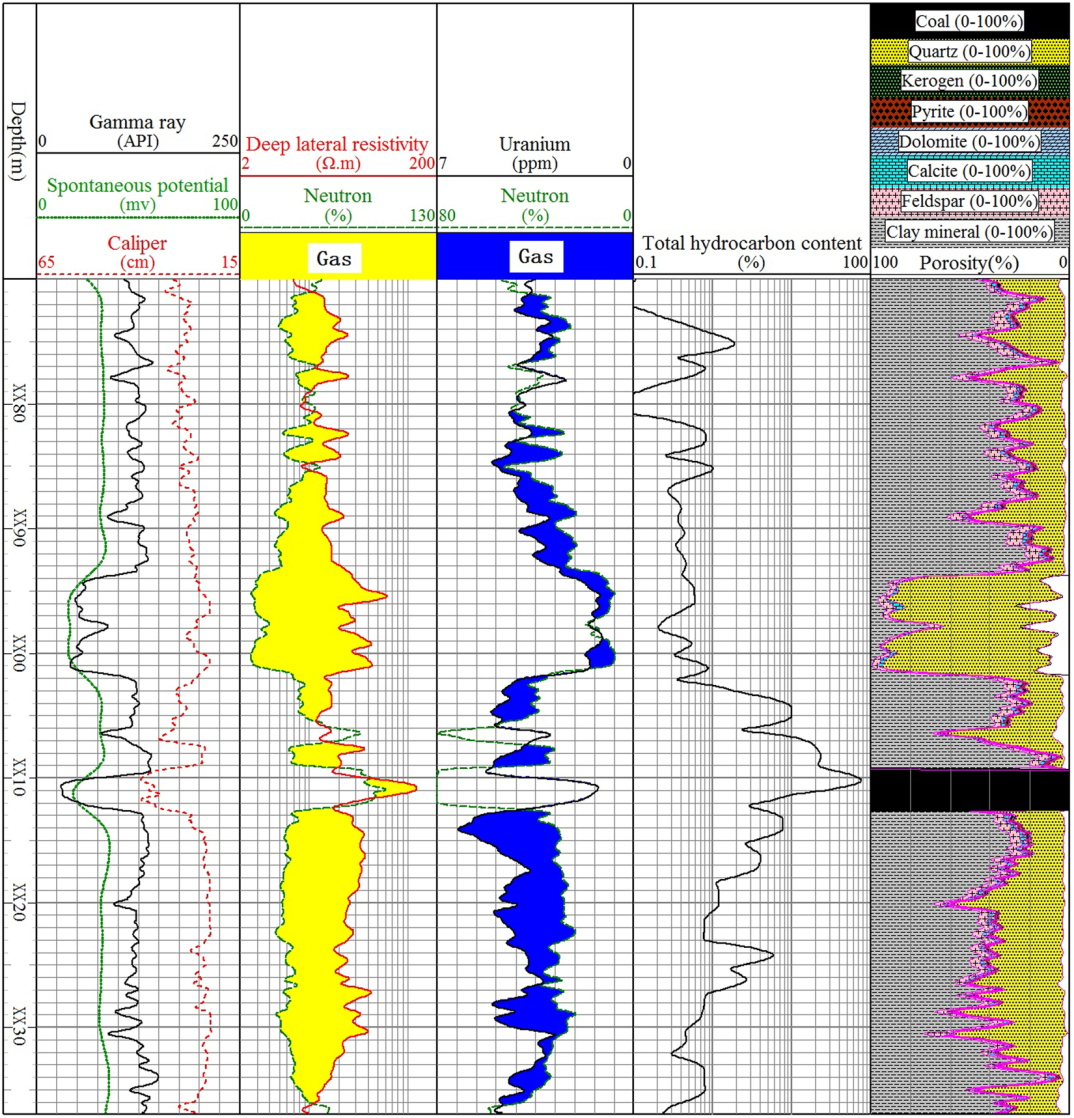
Logging response statistics of mudstone with different total gas content.

Compared with mudstone with total gas content less than 1 m^3^/t, the resistivity and uranium of mudstones with larger total gas content increase, acoustic transit time, neutron and density decrease, and natural gamma ray, amplitude difference of spontaneous potential, thorium and potassium hardly change (Fig. 8). Table 4 summarizes the logging response characteristics of mudstones with total gas content less than 1 m^3^/t and greater than 1 m^3^/t. In terms of the degree of difference, the resistivity and neutron are the most obvious among all these differences, followed by uranium, and the acoustic transit time, thorium and potassium are the least obvious. Therefore, we can use neutrons, resistivity and uranium to identify gas-bearing mudstones.

**Table 4:** Well log response statistics of gas-bearing and without gas-bearing mudstone in Shanxi Formation with 4 wells.

Log data	Total gas content ≥ 1 m^3^/t		Total gas content < 1 m^3^/t	
	Distribution range	Dominant distribution range	Distribution range	Dominant distribution range
GR (API)	85.0–185.2	118.0–140.0	85.0–150.2	116.0–138.0
RT (Ω·m)	14.2–90.2	20.0–50.0	4.1–25.2	15.0–19.0
AC (μs/m)	200.0–320.0	221.0–262.0	198.0–342.4	221.0–250.0
CNL (%)	9.0–37.2	18.0–33.0	20.0–59.2	23.0–35.0
DEN (g/cm^3^)	2.18–2.93	2.44–2.63	2.03–2.74	2.52–2.74
K (%)	0.9–4.2	1.0–2.3	0.6–3.1	1.2–2.2
TH (ppm)	10.0–24.5	16.0–19.0	3.6–23.2	12.5–21.2
U (ppm)	2.8–5.6	3.2–5.0	1.6–4.4	3.0–4.4
ΔSP (mv)	−12.7–16	−9.0–0	−12.4–15	6.0–1.0

### Well log evaluation

Logging evaluation of mudstone reservoirs in the study area includes qualitative and quantitative evaluation. Qualitative evaluation includes analysis of sedimentary environment and effective identification of the relative gas content of mudstone reservoirs at different depths. The quantitative evaluation mainly calculates the gas content, porosity, permeability and brittleness index of mudstone reservoirs. Well logging evaluation is based on conventional logging data from four wells and natural gamma spectroscopy logging data from wells A1, A2, and A4. Only the A4 well collected the total hydrocarbon content curve.

#### Sedimentary environment

**Figure 9:**
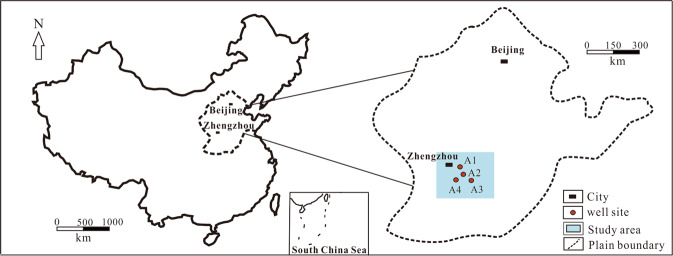
Natural gamma spectroscopy logging interpretation charts for wells A1, A2, and A4. where TH is thorium, K is potassium, U is uranium.

One of the main applications of natural gamma-ray spectroscopy logging is to analyze the sedimentary environment of the formation. Thorium is insoluble in water and transported in suspension. Therefore, the content of thorium in high energy deposition environment is higher than that in low energy deposition environment. The content of uranium in the reducing environment is higher than that in the oxidizing environment, while potassium is opposite to uranium, and its content in the oxidizing environment is higher than that in the reducing environment^36^. The ratio of thorium to potassium (TH/K) is a parameter that can reflect the sedimentary energy. The ratio above 10 is high energy sedimentary environment, 10−6 is sub-high energy sedimentary environment, 6−3 is low-energy sedimentary environment. In addition, according to the logging interpretation chart of Schlumberger (Fig. 9(a)), TH/K can also be used to analyze the type of clay minerals. The ratio is 0.3−0.6 for feldspar, 0.6−1.5 for glauconite, 1.5−2 for mica, 2−3.5 for illite, 3.5−12 for mixed layer clay, and 12−25 for kaolinite^37^. The ratio of strontium to uranium (TH/U) is a parameter reflecting the sedimentary environment, which is high in the oxidizing environment and low in the reducing environment. When TH/U > 7, it indicates the oxidative environment caused by the continental sedimentation, 2 < Th/U ≤ 7 indicates the oxidation-reduction environment caused by the marine-continental transitional facies sedimentation, and Th/U ≤ 2 indicates the reduction environment caused by the marine sedimentation^38^.

Figure 9 shows that the TH/K distribution of the mudstones in the Shanxi Formation in the study area is between 5 and 25, reflecting that the change of sedimentary energy is from high energy to low energy, mainly sub-high energy sedimentary energy; clay mineral is mainly mixed layer clay with a small amount of kaolinite. TH/U is mainly distributed between 2 and 7, reflecting the sedimentary environment mainly belongs to the oxidation-reduction environment caused by the marine-continental transitional facies sedimentation.

#### Gas content

Using the logging curve overlap method, the total gas content of the mudstone below the coal seam is higher than above. Figure 2 is an example of a qualitative evaluation of the relative total gas content of well A4.

According to the core measurement data analysis, the adsorption gas content is positively correlated with clay content and total organic carbon content, and the calculation formula is obtained by multiple regression. The calculated adsorption gas content of the four wells ranges from 0.1−1.4 m^3^/t with an average of 0.8 m^3^/t. Similarly, the core measurement of free gas content is also positively correlated with the adsorbed gas content. The free gas content distribution range of the four wells calculated by the regression formula is 0.2−3.1 m^3^/t, and the average value is 1.3 m^3^/t. The total gas content is the sum of the adsorbed and free gas contents, which ranges from 0.1 to 4.1 m^3^/t in four wells, with an average of 1.6 m^3^/t. The fourth column of Table 4 shows the total gas content distribution range and average for each well.

Figure 3 shows the logging evaluation results of the total gas content of the four wells. The results show that the total gas content of the mudstone near the coal seam is relatively higher in the four wells. The A4 well has the highest total gas content, and its highest point is located below the adjacent coal seam.

#### Porosity and permeability

Compared to core measured data, the use of well log data calculations not only provides continuous depth porosity and permeability but also lower costs. Density, neutron and acoustic transit time can all be used to calculate porosity, but their accuracy is different, especially where the diameter within the wellbore is enlarged. The density is most affected by the diameter expansion in the wellbore, although it can be corrected but only approximated. Since the neutron is greatly affected by the hydrogen index of the formation, the porosity calculated by the neutron in the gas-bearing formation is reduced. Therefore, in comparison, the acoustic transit time is less affected by the diameter and gas content in the wellbore, and the porosity calculated using the corrected acoustic transit time is the most reasonable.

The porosity of the gas-bearing mudstones calculated by the acoustic transit time in the four wells ranged from 0.7 to 6.2% with an average of 2.8%. The permeability is calculated according to the formula obtained from the regression analysis of the core experimental data, which is a exponential function relationship with the porosity. By calculating its distribution range is 0.0012−0.1040 mD, the average value is 0.0235 mD. The second and third columns of Table 5 show the porosity and permeability values for each well. It should be noted that due to the lack of electrical imaging logging data, the calculated porosity is only the matrix porosity and permeability of the mudstone. The porosity and permeability of the fracture have not been studied in this paper.

**Table 5:** Logging evaluation of gas-bearing mudstone reservoirs in the Shanxi Formation with four wells.

Borehole ID	Total gas content (m^3^/t)		Porosity (%)		Permeability (mD)		Brittleness index (%)	
	range	Average	range	Average	range	Average	range	Average
A1	0.1–2.6	1.4	0.8–5.5	3.1	0.0032−0.0160	0.0112	11.5−49.7	29.1
A2	0.8–2.5	1.6	1.1–4.9	2.2	0.0027−0.0153	0.0108	10.2−57.2	35.2
A3	0.1–2.0	1.2	0.7–3.7	2.0	0.0012−0.0130	0.0050	18.9−55.8	42.9
A4	0.3–4.1	2.2	1.1–6.2	3.8	0.0130−0.1040	0.0670	8.4−68.1	45.2
Total	0.1–4.1	1.6	0.7–6.2	2.8	0.0012−0.1040	0.0235	8.4−68.1	38.1

#### Brittleness index

Since the content of adsorbed gas in mudstone reservoirs is much larger than that in sandstone reservoirs, only large scale hydraulic fracturing can achieve higher yields and longer production periods for gas-bearing mudstone reservoirs. The brittleness index indicates the degree of difficulty in fracturing and can also reflect the degree of cracking in the reservoir after fracturing. Therefore, as with shale, the evaluation of rock brittleness index is also one of the important parameters for evaluating the fractureability of gas-bearing mudstone reservoirs. Based on the ratio of brittle mineral content to total mineral content in mudstone, the brittleness index of the four wells ranges from 8.4 to 68.1% with an average of 38.1%. These data are shown in the last column of Table 5.

## Conclusions

The gas-bearing mudstone reservoirs formed by the marine-continental transitional sedimentation environment are common in the Shanxi formation in the south of North China Plain. The organic matter has a high degree of thermal evolution, and the kerogen of the reservoir releases only gas without oil. The values of various characteristic parameters of gas-bearing mudstone in Shanxi formation of four wells are as follows: average thickness is 55.7 m, average total gas content is 1.6 m^3^/t, average porosity is 2.8%, average permeability is 0.0235 mD, and average brittleness index is 38.1%. The A4 well has the largest total gas content, with a distribution range of 0.3−4.1 m^3^/t and an average of 2.2 m^3^/t. It is expected that high yields can be obtained, and it also indicates that the gas-bearing mudstone reservoirs of the Shanxi formation in A4 well area have exploration prospects. These data also show that the gas-bearing mudstone reservoir has the same exploitation potential as shale gas reservoir. It deserves more attention.

## Data Availability

The authors declare that all materials and data in this paper are available, and others can replicate and build upon the authors’ published claims.
