# Publisher Correction: Reservoir characteristics and logging evaluation of gas − bearing mudstone in the south of North China Plain

**DOI:** 10.1038/s41598-020-76387-6

**Published:** 2020-11-05

**Authors:** Liang Liu, Heping Pan, Zhenzhou Lin, Shihui Zhang, Zhen Qin, Jianwei Li, Guoshu Huang, Lei Wang, Dong Li

**Affiliations:** 1 Institute of Geophysics and Geomatics, China University of Geosciences (Wuhan), Wuhan, 430074 China; 2 Institute of Geophysical and Geochemical Exploration, CAGS, Langfang, 065000 China; 3 School of Geophysics and Measurement-Control Technology, East China University of Technology, Nanchang, 330013 China; 4 Well Logging Company of Sinopec North China Petroleum Engineering Company, Zhengzhou, 450007 China

Correction to: *Scientific Reports* 10.1038/s41598-020-65325-1, published online 29 May 2020

This Article contains an error in the order of the Figures. Figures 1, 2, 3, 4, 6, 7, 8 and 9 are published as Figures 9, 8, 7, 6, 4, 3, 2 and 1 respectively. The correct Figures 1, 2, 3, 4, 6, 7, 8 and 9 appear below. The Figure legends are correct.

**Figure 1:**
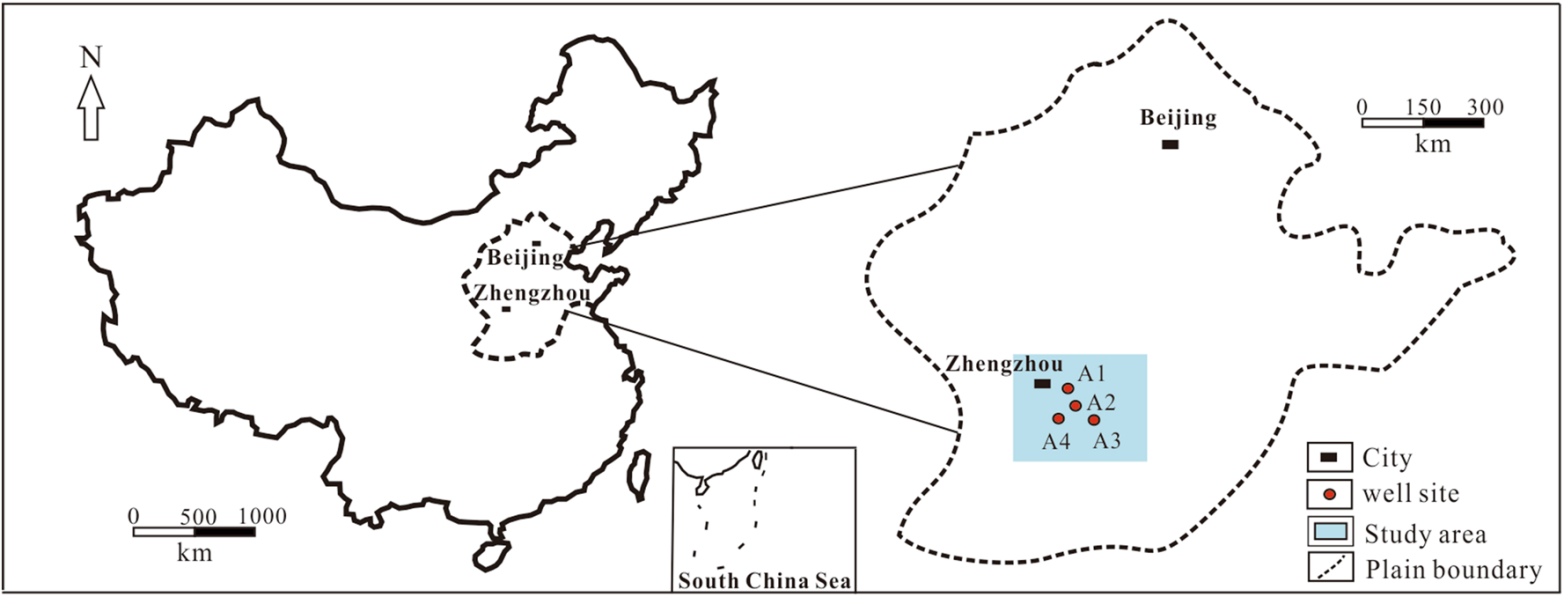
Location of 4 wells in the study area.

**Figure 2:**
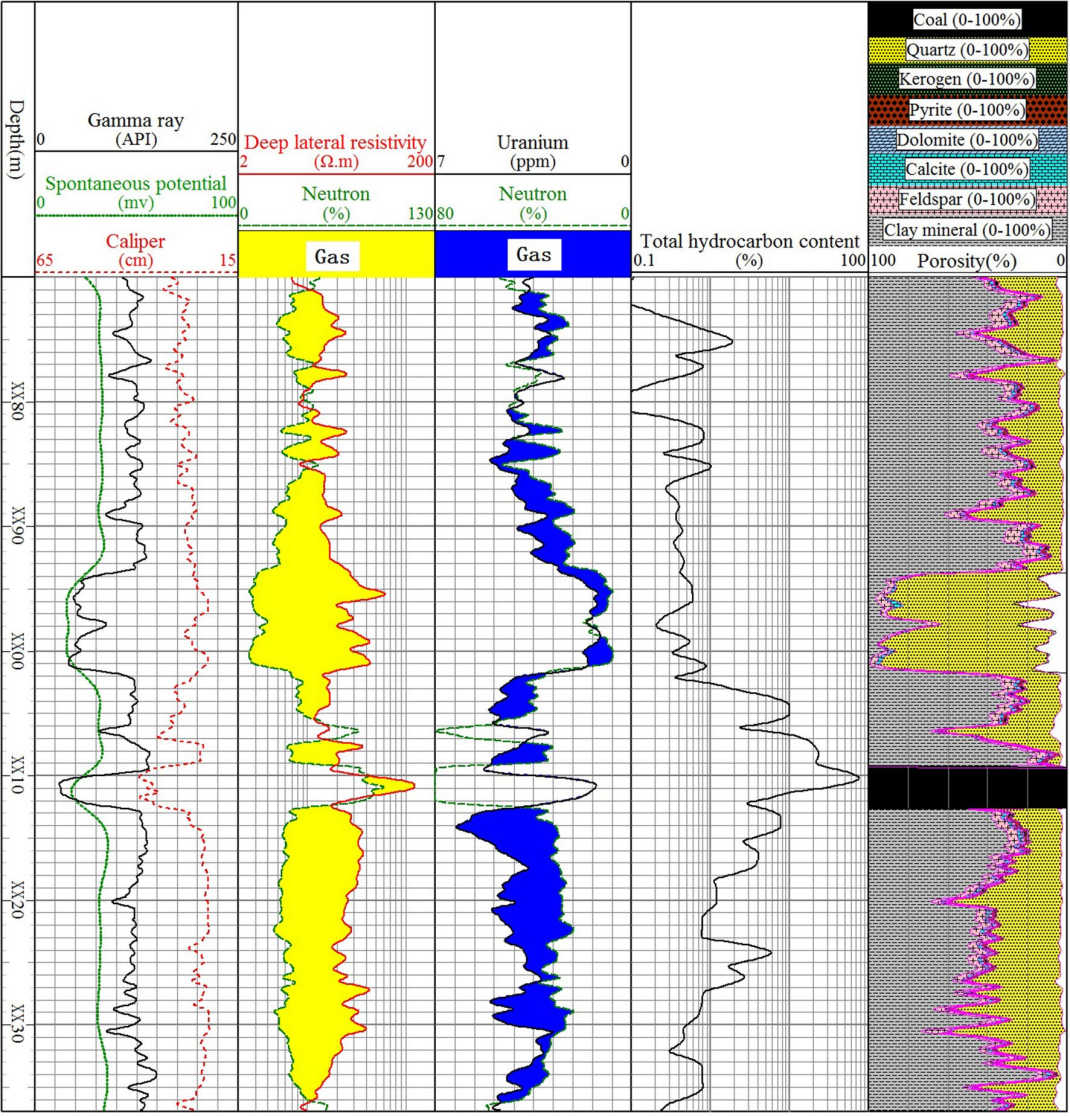
Qualitative evaluation of the relative gas content of mudstone in Shanxi formation of well A4.

**Figure 3:**
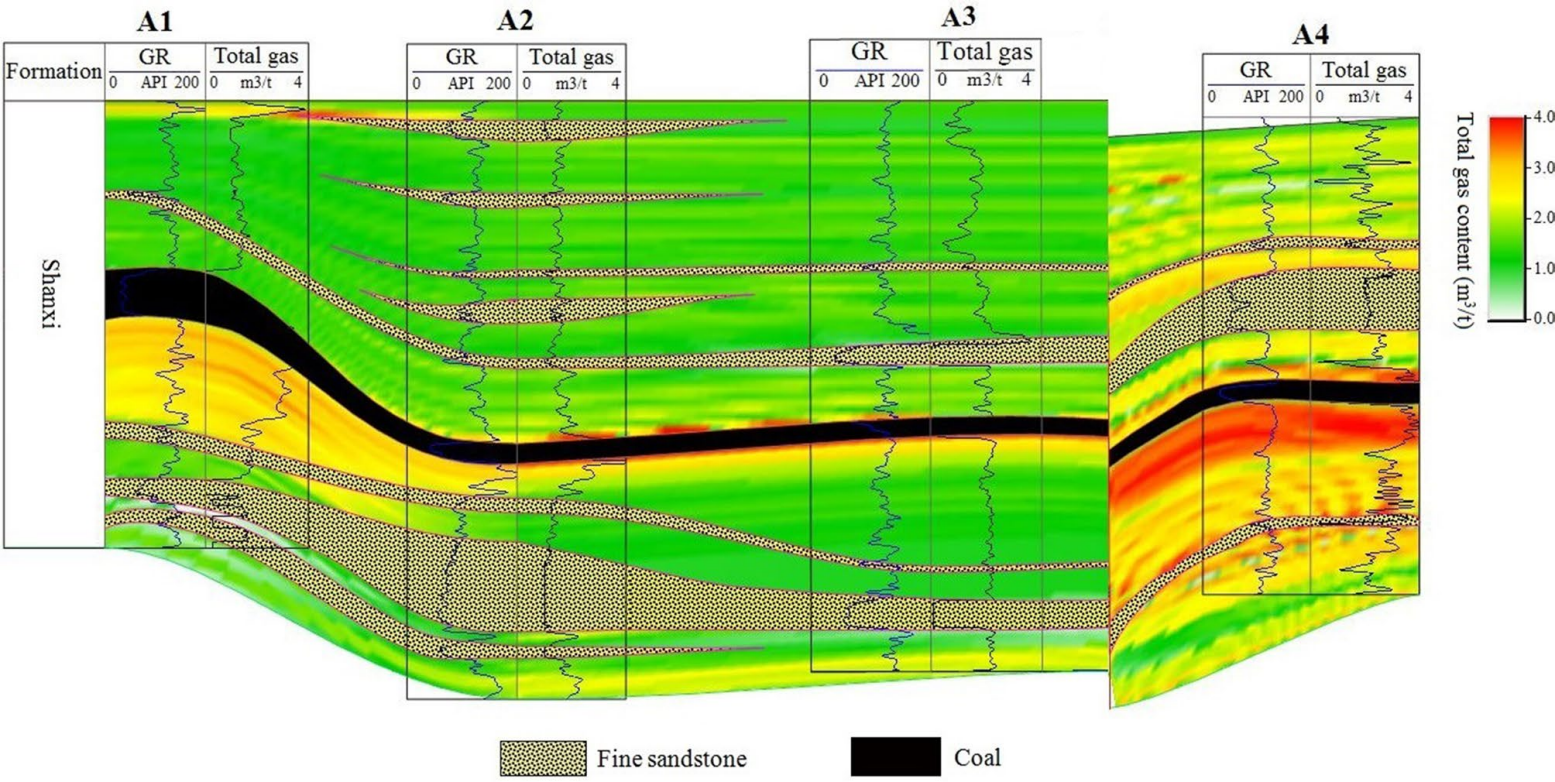
Logging evaluation of total gas content of mudstone in Shanxi formation of four wells.

**Figure 4:**
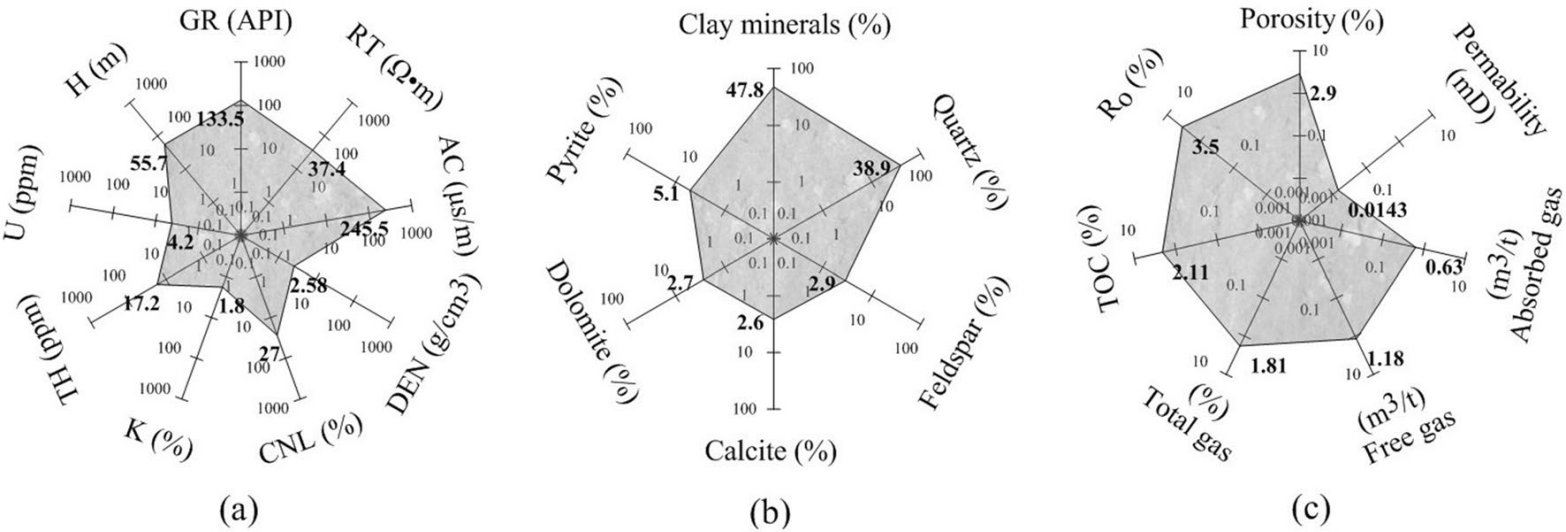
Average values of various characteristics of gas bearing mudstones in Shanxi formation of four wells. Response characteristics from logging data (**a**). Mineral characteristics (**b**), geophysical and geochemical characteristics (**c**) from core measurements. Where GR is the natural gamma ray, RT is the deep lateral resistivity, AC is the acoustic transit time, DEN is the density, CNL is the neutron, K is the potassium, TH is the thorium, U is the uranium, H is the thickness, TOC is the total organic carbon content, and Ro is the vitrinite reflectance.

**Figure 6:**
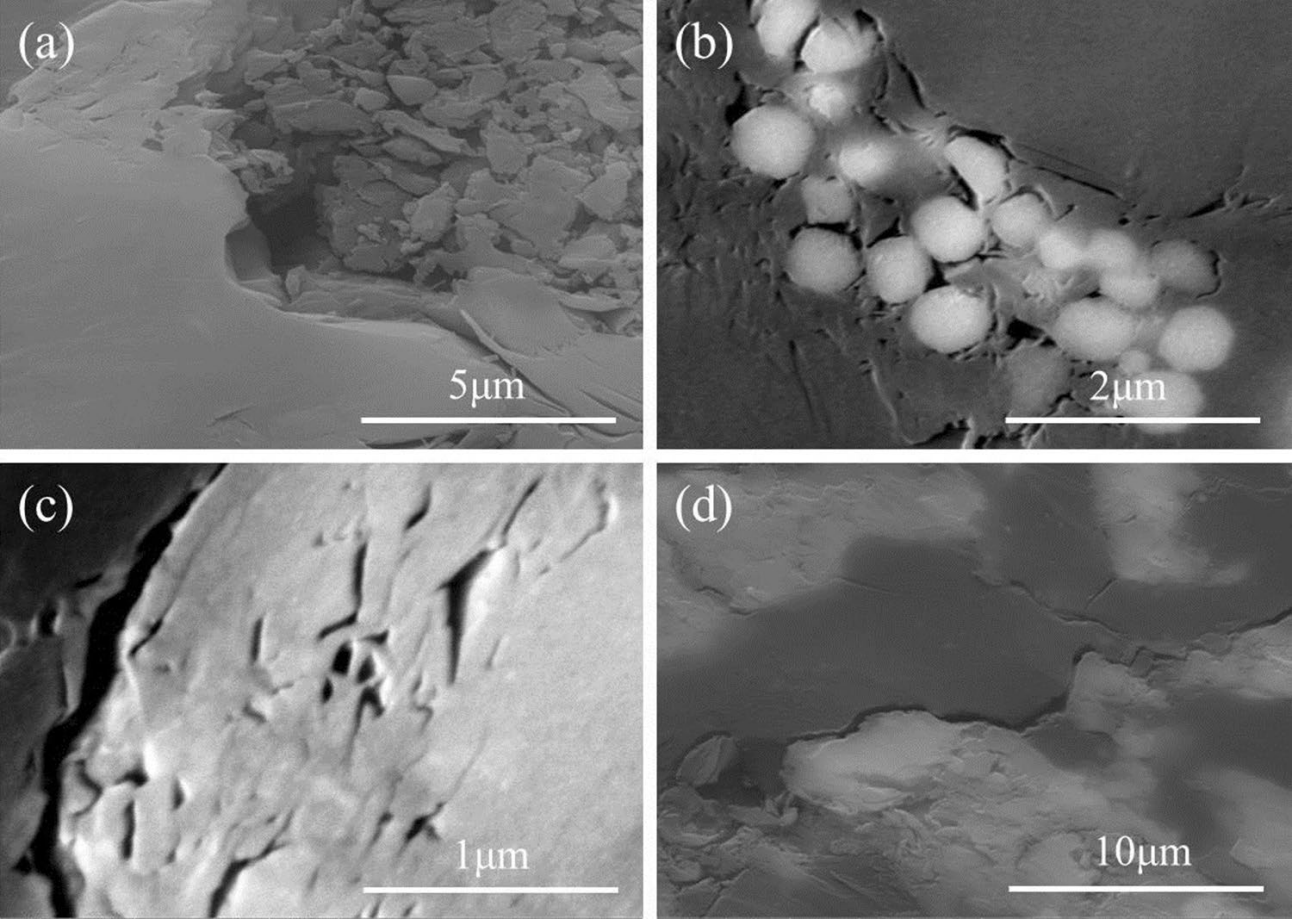
SEM images of intergranular pore (**a**,**b**), intragranular pore (**c**) and microcrack (**d**) in the gas−bearing mudstone reservoir of well A2.

**Figure 7:**
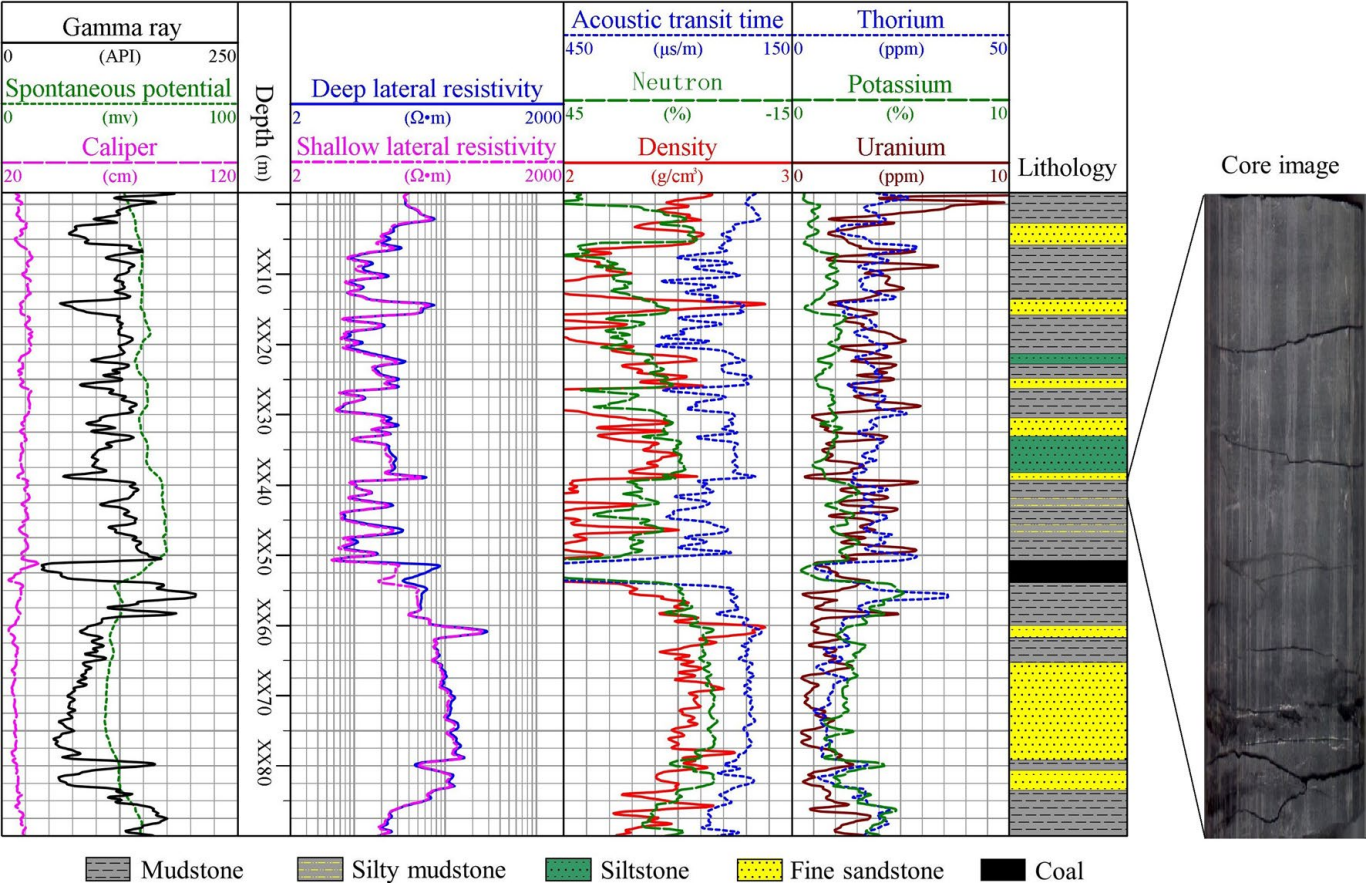
Well logging response of the Shanxi formation in the well A2.

**Figure 8:**
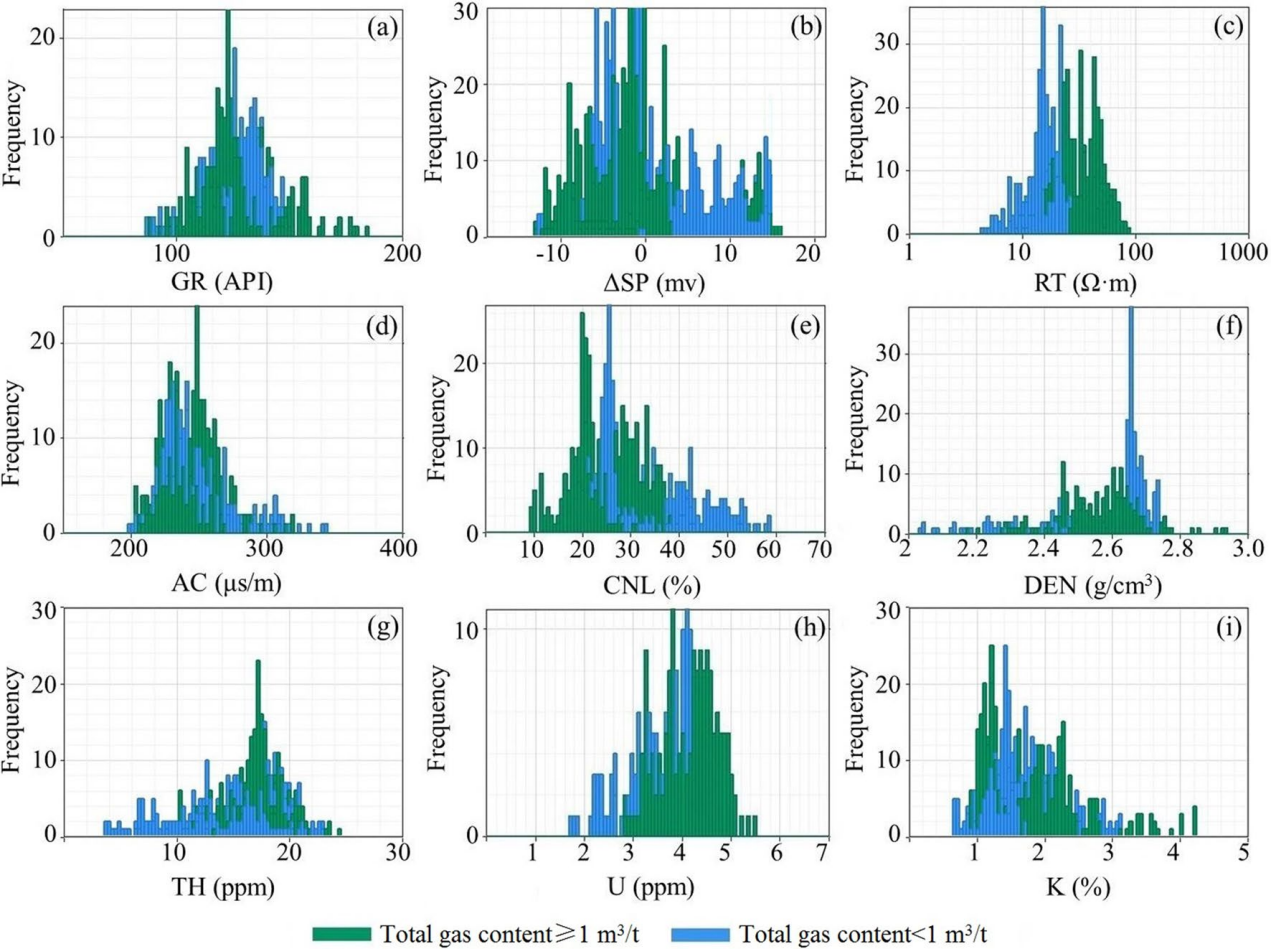
Logging response statistics of mudstone with different total gas content.

**Figure 9:**
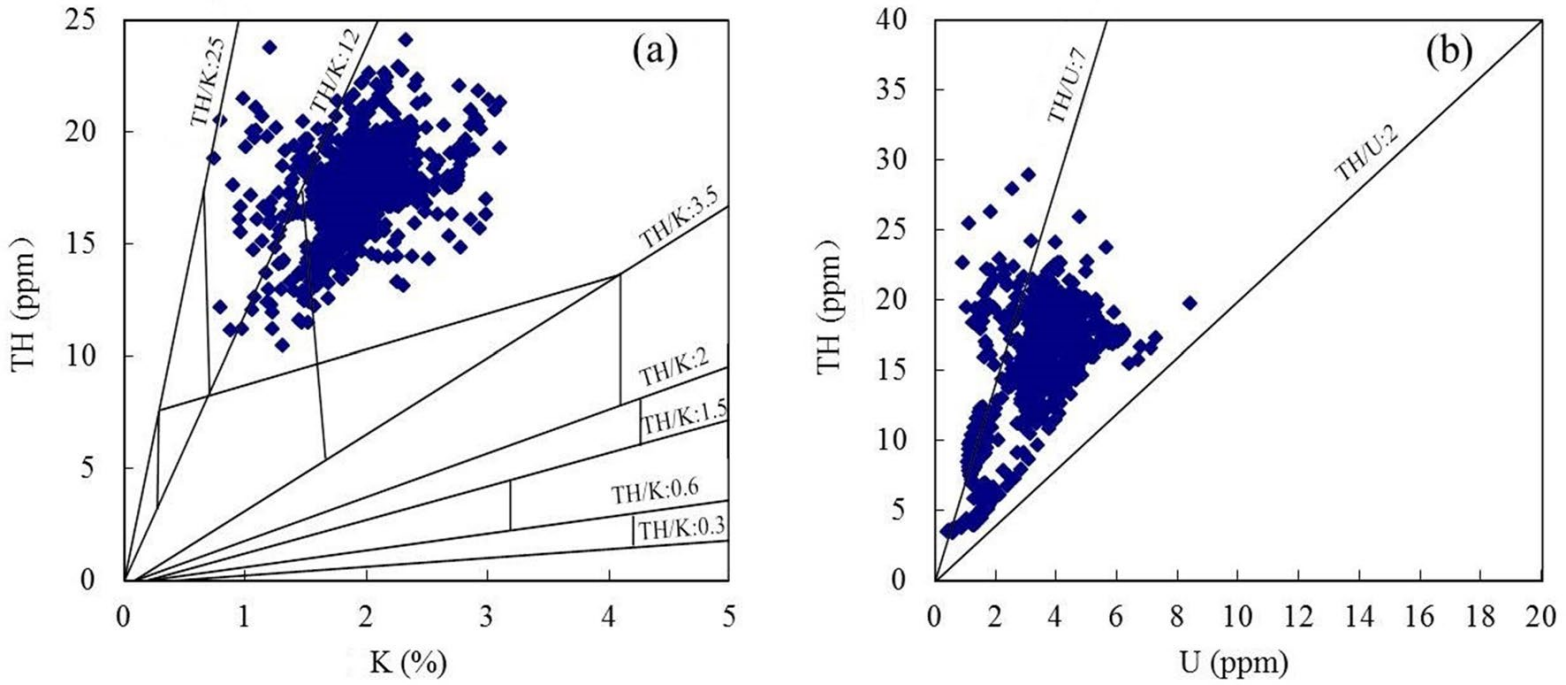
Natural gamma spectroscopy logging interpretation charts for wells A1, A2, and A4. where TH is thorium, K is potassium, U is uranium.

